# Multiphase MRI radiomics model for predicting microvascular invasion in HCC: Development and clinical validation

**DOI:** 10.1016/j.iliver.2025.100165

**Published:** 2025-04-26

**Authors:** Yue Peng, Songxiong Wu, Bing Xiong, Fuqiang Chen, Nazar Zaki, Ruodai Wu, Wenjian Qin

**Affiliations:** a Shenzhen Institutes of Advanced Technology, Chinese Academy of Sciences, Shenzhen 518055, China; b University of Chinese Academy of Sciences, Beijing 100049, China; c Department of Radiology, Shenzhen University General Hospital, Shenzhen University, Shenzhen 518000, China; d Department of Computer Science and Software Engineering, College of Information Technology, United Arab Emirates University (UAEU), Al Ain 15551, United Arab Emirates

**Keywords:** Hepatocellular carcinoma, Microvascular invasion, Multi-phase MRI

## Abstract

**Background and aims:**

Accurate preoperative prediction of microvascular invasion (MVI) in hepatocellular carcinoma (HCC) is crucial for treatment planning. This study aimed to develop and validate a multi-phase magnetic resonance imaging (MRI)-based radiomics model for predicting MVI in HCC patients.

**Methods:**

This retrospective study included 110 HCC patients (training: *n* = 77; validation: *n* = 33) who underwent preoperative multi-phase MRI. Radiomics features were extracted from four MRI phases (non-contrast, arterial, portal, and hepatobiliary). Feature selection was performed using least absolute shrinkage and selection operator regression, and five machine learning classifiers were evaluated. Model performance was assessed using standard metrics including area under the receiver operating characteristic curve (AUC), sensitivity, specificity, and accuracy.

**Results:**

The four-phase radiomics model with logistic regression classifier showed optimal performance in both the training (AUC = 0.896; 95% confidence interval, 0.792–0.963) and validation cohorts (AUC = 0.889, 95% confidence interval, 0.781–0.982), outperforming the single-phase (AUC = 0.789), two-phase (AUC = 0.815), and three-phase models (AUC = 0.848) in the validation cohort. In the validation cohort, the model achieved balanced performance with sensitivity, specificity, accuracy, and precision all reaching 0.857.

**Conclusions:**

The multi-phase MRI-based radiomics model significantly improves MVI prediction accuracy in HCC patients. This non-invasive approach could enhance preoperative assessment and treatment planning.

## 1 Introduction

Hepatocellular carcinoma (HCC) ranks among the most prevalent and lethal malignant neoplasms worldwide, presenting a significant global health challenge.^1^, ^2^, ^3^ A critical determinant of patient outcome in HCC is microvascular invasion (MVI), which is characterized by infiltration of tumor cells into the small vessels of the surrounding liver tissue. MVI status profoundly influences therapeutic decision-making, including determination of surgical resection margins, selection between liver transplantation and resection, and the need for adjuvant therapy.^4^^,^^5^ The reported incidence of MVI in HCC patients ranges substantially from 15% to 57%.^6^^,^^7^ Despite its crucial role in clinical decision-making, accurate preoperative prediction of MVI status remains an unmet challenge in contemporary oncology. Conventional imaging modalities frequently underestimate the presence of MVI, potentially resulting in suboptimal treatment strategies.^8^ While magnetic resonance imaging (MRI) has emerged as a promising diagnostic tool for MVI prediction, existing research has predominantly focused on single-phase imaging features, which may inadequately capture the complex pathophysiology of vascular invasion in HCC.^9^ Recent technological advances in medical imaging and radiomics have created unprecedented opportunities for comprehensive tumor characterization. Multi-phase MRI sequences offer distinct and complementary insights into tumor biology: the non-contrast phase reveals fundamental tumor morphology; the arterial phase delineates tumor vascularity patterns; the portal phase demonstrates contrast washout characteristics; and the hepatobiliary phase provides crucial information about functional hepatic tissue properties.^10^ However, the potential of integrated multi-phase MRI feature analysis for MVI prediction remains largely unexplored.

Recent advances in machine learning algorithms have demonstrated remarkable potential in medical image analysis and pattern recognition. While various classifiers—including logistic regression, support vector machine, and random forest—have shown distinct capabilities in processing radiomics features, the optimal approach for MVI prediction using multi-phase MRI radiomics features has not been determined.^11^, ^12^, ^13^ Although several studies have explored prediction of MVI using radiomics features, few have examined the combined value and relative contributions of different MRI phases to diagnostic accuracy. The significance of MVI as an indicator of poor prognosis in HCC patients is well-established. Hu et al.^14^ demonstrated through comprehensive analysis that its presence in patients undergoing surgical resection significantly correlates with increased recurrence and decreased overall survival. The predictive accuracy of traditional imaging approaches for MVI is limited, as confirmed by Hong et al.^15^ in their systematic review and meta-analysis. Wu et al.^16^ found that even contrast-enhanced imaging achieved only moderate sensitivity (62%–78%) for MVI detection. Recent investigations into individual MRI phases for MVI prediction have shown varying degrees of success: Liu et al.^17^ achieved an area under the curve (AUC) of 0.71 using T1-and T2-weighted imaging features; Kim et al.^18^ developed a machine learning model based on arterial enhancement patterns with 76% accuracy; Cheng et al.^19^ reported 73% sensitivity using a radiomics nomogram based on portal phase characteristics; and Li et al.^20^ achieved an AUC of 0.77 using gadoxetic acid-enhanced MRI features. The emergence of radiomics has introduced new possibilities in HCC analysis. Zhang et al.^21^ developed a deep learning-based model using multi-phase MRI for MVI prediction, while Ji et al.^22^ applied radiomics to predict early recurrence. However, most studies have focused on single-phase imaging analysis, with limited exploration of multi-phase integration. Li et al.^20^ demonstrated an 8% improvement in prediction accuracy by combining arterial and hepatobiliary phases, while Zhang et al.^23^ achieved an AUC of 0.82 in multi-center validation using integrated multi-phase MRI features. Despite these advances, several limitations exist in current research: most studies rely on single-phase imaging, potentially missing complementary information from other phases.^16^ The relative contribution of different MRI phases to MVI prediction remains unclear, and few studies have systematically evaluated the integration of all available MRI phases.^24^ Furthermore, there is limited external validation of multi-phase models in clinical settings.^25^ These limitations highlight the need for a comprehensive multi-phase approach to MVI prediction, which forms the basis of our current study. Despite these advances, current research has several limitations: (1) predominant reliance on single-phase imaging, potentially overlooking complementary information from other phases; (2) unclear relative contributions of different MRI phases to MVI prediction; (3) insufficient systematic evaluation of integrated MRI phases; and (4) limited external validation of multi-phase models in clinical settings.

To address these gaps, this study aims to:

(1) Systematically analyze and compare the diagnostic value of individual MRI phases (pre-enhanced T1-weighted axial imaging, arterial, portal, hepatobiliary) for MVI prediction.
(2) Develop and validate a multi-phase radiomics model that integrates features from all available MRI phases to improve predictive accuracy.
(3) Evaluate the clinical applicability of the integrated approach through external validation and multi-classifier analysis.

Our contributions are summarized as follows:

• A systematic comparison of individual MRI phases' diagnostic value for MVI.
• An optimized multi-phase radiomics model with improved accuracy.
• Clinical validation of the model's reliability and generalizability.

## 2 Methods

**Fig. 1:**
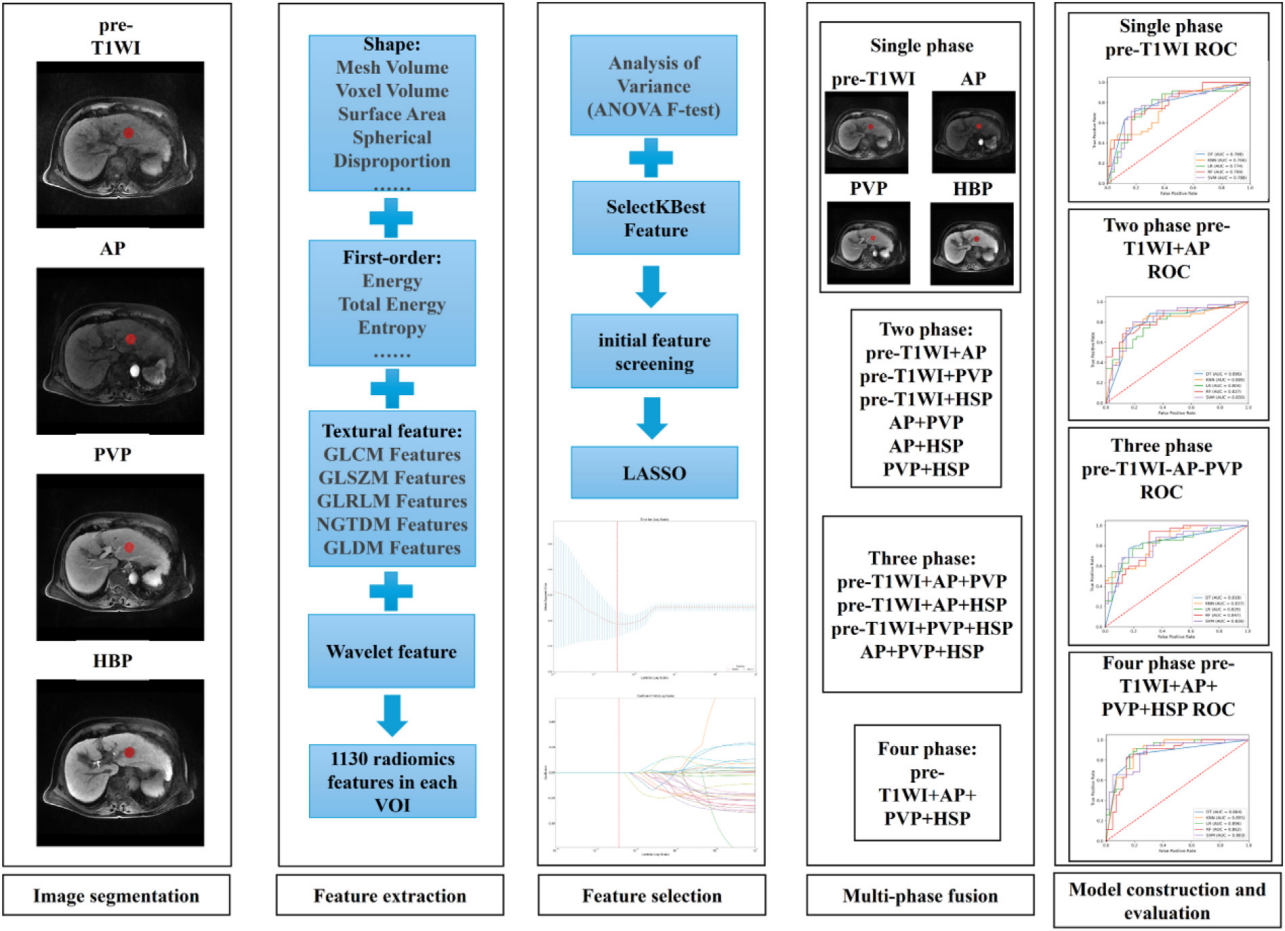
Flowchart of radiomics analysis.

**Fig. 2:**
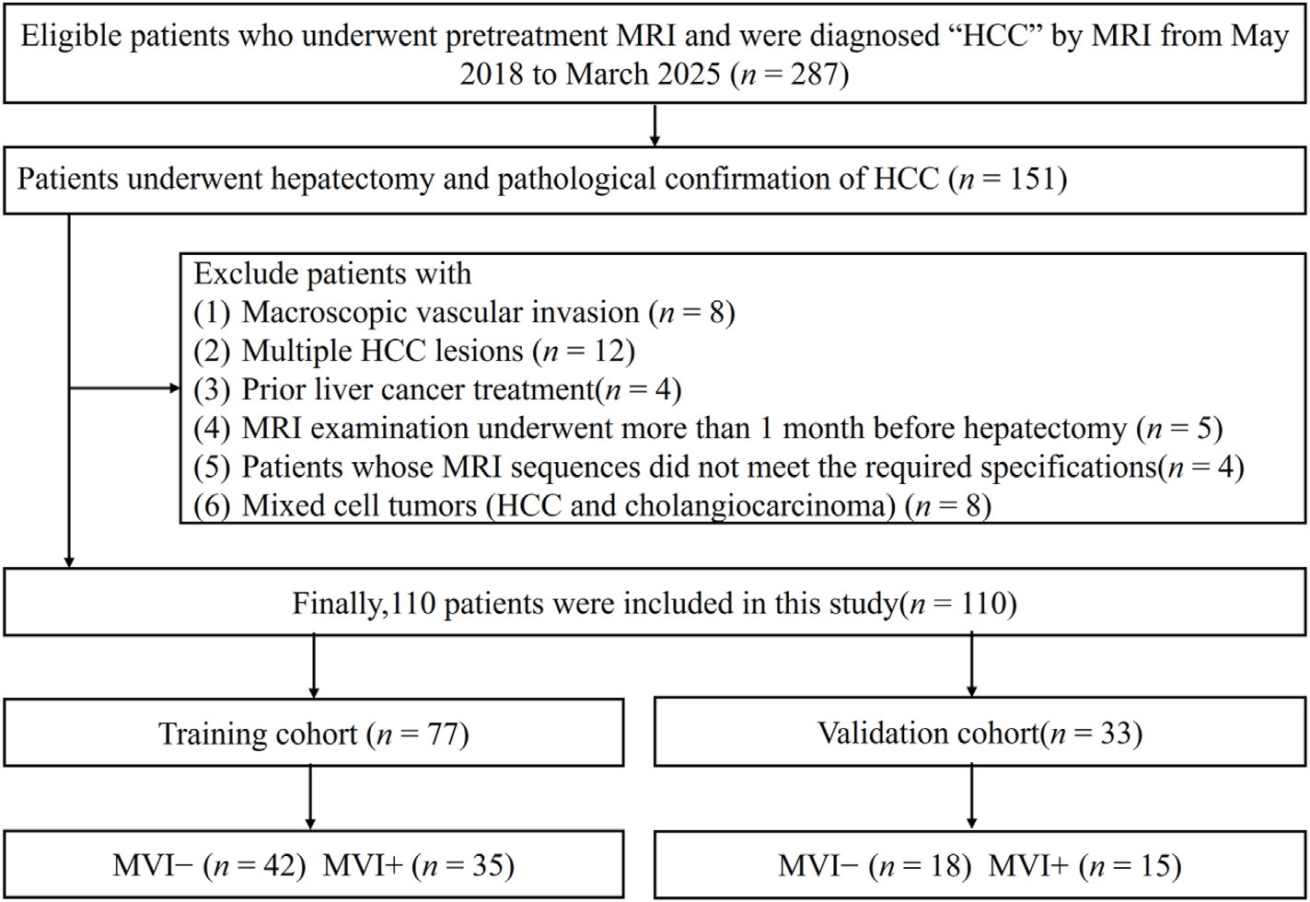
Study flowchart. HCC, hepatocellular carcinoma; MVI, microvascular invasion; MVI−, MVI-negative; MVI+, MVI-positive.

This retrospective study received institutional ethics review board approval. The requirement for informed consent was waived because the data were anonymized. Patients with a single histologically confirmed primary HCC who underwent gadolinium ethoxybenzyl-diethylenetriaminepentaacetic acid-enhanced MRI within 30 days of curative hepatectomy were eligible for inclusion. Those with a history of previous liver cancer treatment and patients who had undergone anticancer therapy before surgery were excluded. We also excluded patients whose MRI phases did not meet required quality specifications. The final study population consisted of 110 HCC patients. The radiomics analysis workflow, including image segmentation, feature extraction, feature selection, multi-phase fusion and model classification, is illustrated in Fig. 1. Using a randomized 7:3 ratio, patients were divided into a training cohort (TC, *n* = 77; 68 males and 9 females) and a validation cohort (VC, *n* = 33; 28 males and 5 females). A study flowchart is presented in Fig. 2.

### 2.1 MRI acquisition

**Fig. 3:**
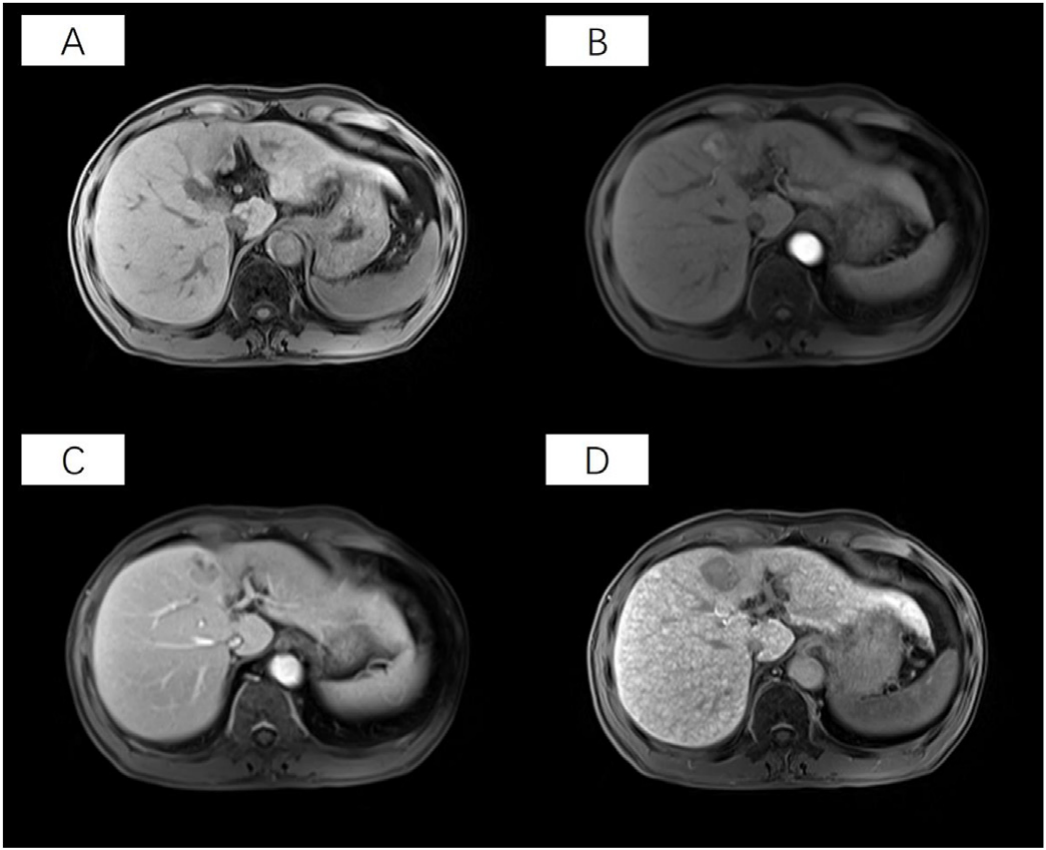
Magnetic resonance imaging phase images of a 49-year-old patient. (A) Pre-enhanced T1-weighted imaging before contrast administration (T1W1). (B) Hepatobiliary phase (HBP). (C) Arterial phase (AP). (D) Portal venous phase (PVP).

Gadolinium ethoxybenzyl-diethylenetriaminepentaacetic acid-enhanced MRI was performed using a 3.0 T system (Siemens, Munich, Germany). The protocol comprised sequential phases: pre-enhanced T1-weighted axial imaging (pre-T1WI), arterial phase (AP) imaging, portal venous phase (PVP) imaging, and hepatobiliary phase (HBP) imaging. Dynamic imaging was performed after intravenous administration of gadoxetic acid (0.1 mL/kg) at a flow rate of 1 mL/s. The dynamic post-contrast three-dimensional (3D) T1-weighted volumetric interpolated breath-hold examination (VIBE) captured the arterial phase at 20–30 s, portal venous phase at 60–70 s, and hepatobiliary phase at 20 min after injection. Complete scanning parameters are detailed in Table 1. Representative multi-phase MRI phases from a 49-year-old male patient, including pre-T1WI, AP, PVP, and HBP images, are illustrated in Fig. 3.

**Table 1:** Magnetic resonance imaging phase and scanning parameters.

Phases	TR (ms)	TE (ms)	Flip angle	Thickness (mm)	FOV (mm)	Matrix
Pre-T1WI	4.0	2.5	9	3	285 × 380	320 × 260
AP	2.7	1.09	9	3	285 × 380	320 × 260
PVP	2.7	1.09	9	3	285 × 380	320 × 260
HBP	4.3	1.29	9	3	285 × 380	320 × 260

AP, arterial phase; HBP, hepatobiliary phase; FOV, field of view; pre-T1WI, pre-enhanced T1-weighted imaging; PVP, portal venous phase; TE, echo time; TR, repeat time; mm, millimeters; ms, milliseconds.

MVI was graded according to the 2022 Guidelines for the Diagnosis and Treatment of Primary Liver Cancer in China: M0 (no MVI detected), M1 (≤5 MVIs, which occur in proximal non-neoplastic adjacent liver tissues), or M2 (>5 MVIs in the proximal or MVIs occurring in distal non-neoplastic adjacent liver tissues).^26^ Patients graded as M0 were classified as MVI-negative (MVI−) and those as M1 or M2 as MVI-positive (MVI+).

### 2.2 Tumor segmentation and radiomic features extraction

Tumor segmentation was performed on axial slices from all four MRI phases using ITK-SNAP software (www.itksnap.org) by a board-certified abdominal radiologist, which was then verified by a senior radiologist with over 10 years of abdominal imaging expertise. To optimize MRI texture measurements, an intensity outlier exclusion method was implemented as described by Collewet et al.,^27^ removing voxels within the tumor region whose intensities fell outside μ ± 3σ (where μ and σ denote the mean and standard deviation of tumor region intensities, respectively).

Feature extraction was performed using the Pyradiomics toolkit (v3.0.1). Prior to feature extraction, images underwent preprocessing with a normalization ratio of 50 and were resampled using a BSpline interpolator to match the mean spacing of the training dataset. The extracted features encompassed multiple categories: first-order statistics, shape-based features (both 2D and 3D), texture matrices (GLCM, GLSZM, GLRLM, NGTDM, and GLDM), Laplacian of Gaussian-filtered features (σ = 1.0, 3.0, 5.0), and wavelet-decomposition features (using coiflet 1 function). All features were extracted using default parameter settings as specified in the Pyradiomics documentation. The final feature set comprised 1130 features extracted from each imaging phase (pre-T1WI, AP, PVP, and HBP). Multi-phase feature integration was achieved through direct concatenation of features from individual phases to create a comprehensive feature matrix. Further details are provided in the Supplementary Materials.

### 2.3 Radiomics feature selection

Feature selection was implemented through a two-stage process. Initially, features underwent preliminary screening using the Variance Analysis F-test (f_classif) from scikit-learn. This test evaluated each radiomic feature's discriminative power for HCC MVI by calculating F-statistics, which measure the ratio of between-group to within-group variance. Features yielding higher F-values demonstrated greater discriminative capability between categories. The SelectKBest algorithm was then applied to identify the top 100 most statistically significant features. Following this, we calculated the chi-squared values for the features using the chi2 function from the feature selection library and applied SelectKBest again, this time with k = 30, to further narrow down the features to the top 30 most statistically significant features.

Subsequently, feature refinement was performed using the Least Absolute Shrinkage and Selection Operator (LASSO) with cross-validation. LASSO, implemented through LassoCV from scikit-learn, incorporates L1 regularization into its loss function to control model complexity and perform automatic feature selection by reducing irrelevant feature coefficients to zero. The optimal regularization parameter (alpha) was determined through 10-fold cross-validation, and only features retaining non-zero coefficients were included in the final feature set. Further details are provided in the Supplementary Materials.

### 2.4 Model construction and evaluation

The key features selected in the previous steps were applied to five machine learning classifiers, including decision tree (DT), K-nearest neighbors (KNN), logistic regression (LR), random forest (RF), and support vector machine (SVM), to construct traditional radiomics models for predicting the MVI status of HCC. We systematically compared the performance of these classifiers to identify the most effective model for HCC MVI prediction. The study group was randomly divided into training and validation cohorts at a ratio of 7:3. The model parameters were then optimized in the training cohort using a five-fold cross-validation scheme. In the cross-validation process, the four-fold data were used as the training dataset, and the one-fold data were used as the validation dataset, which was repeated five times, and then the predicted probability of the five-fold validation data was used as a whole to evaluate the performance of the model. During independent testing, we evaluated model performance with the mean of the predicted probabilities of all models produced by cross-validation. Additionally, we compared the performance of single-phase, two-phase, three-phase, and four-phase radiomic features for HCC MVI prediction using the five classifiers.

The LR, SVM, RF, and KNN models were implemented using the scikit-learn Python library (version 3.7). Each classifier was trained on the selected features, with hyperparameter tuning performed via grid search and five cross-validation to optimize performance in the training cohort.

The predictive efficacy of the models was evaluated using receiver operating characteristic (ROC) analysis. The area under the ROC curve (AUC) was calculated to assess the overall model discriminative ability. Additionally, we computed several performance metrics, including sensitivity, specificity, accuracy, precision, and F1 score, to evaluate the models' ability to correctly classify HCC MVI status. These metrics provide a comprehensive assessment of model performance across different aspects, including both false positive and false negative rates.

### 2.5 Statistical analysis

The continuous data are presented as mean ± standard deviation, and categorical data are presented as percentages. To assess and compare the predictive performance of different models, ROC curves for all models were analyzed using MedCalc software (version 12.7.0). The AUC values of the models were compared using the DeLong method. Additional analyses were conducted using the scipy module in Python. The Student's *t*-test was used to compare continuous variables with a normal distribution; the Mann–Whitney U test was to compare those with a non-normal distribution. Categorical variables were compared using the chi-square test. A two-tailed *p*-value <0.05 was considered significant.

## 3 Results

### 3.1 Patient characteristics

Patient characteristics in the training and validation cohorts are described in Table 2. Gender, age, Alpha-Fetoprotein(ATP), nonsmooth tumor margin, and maximum 2D diameter did not significantly differ between the cohorts. Clinical variables included nonsmooth tumor margins and tumor size parameters (quantified via radiomics as Maximum 2D diameter), alongside ATP-related metabolic features.

**Table 2:** Patient characteristics in the training and validation cohorts.

Characteristics	Training cohort (*n* = 77)			Validation cohort (*n* = 33)			*p-*inter
	MVI− (*n* = 42)	MVI+ (*n* = 35)	*p-*intra	MVI− (*n* = 18)	MVI+ (*n* = 15)	*p-*intra	
Age^a^	55.38 ± 10.36	52.57 ± 9.67	0.226	52.77 ± 9.59	55.33 ± 14.33	0.561	0.941
Gender (%)			0.517			0.790	0.617
Male	38 (90.5)	30 (85.7)		15 (83.3)	13 (86.7)		
Female	4 (9.5)	5 (14.3)		3 (16.7)	2 (13.3)		
ATP (%)			0.197			0.197	0.082
≥20 ng/mL	19 (24.7)	21 (27.3)		7 (21.2)	9 (27.3)		
<20 ng/mL	23 (29.9)	14 (18.1)		11 (33.4)	6 (18.1)		
Nonsmooth tumor margin (%)			0.653			0.990	0.709
Absent	28	25		12	10		
Present	14	10		6	5		
Maximum 2D diameter^a^	54.68 ± 34.21	55.06 ± 26.80	0.957	45.55 ± 22.48	19.69 ± 15.65	0.552	0.780
MVI stage							
M0	42	0		18	0		1.000
M1	0	30		0	12		
M2	0	5		0	3		

AFP, alpha fetoprotein; M0, no MVI detected; M1, ≤5 MVIs detected; M2, >5 MVIs detected; MVI−, MVI-negative group; MVI+, MVI-positive group.

a Data are mean ± standard deviation.

### 3.2 Performance of single-phase models in predicting MVI status

**Fig. 4:**
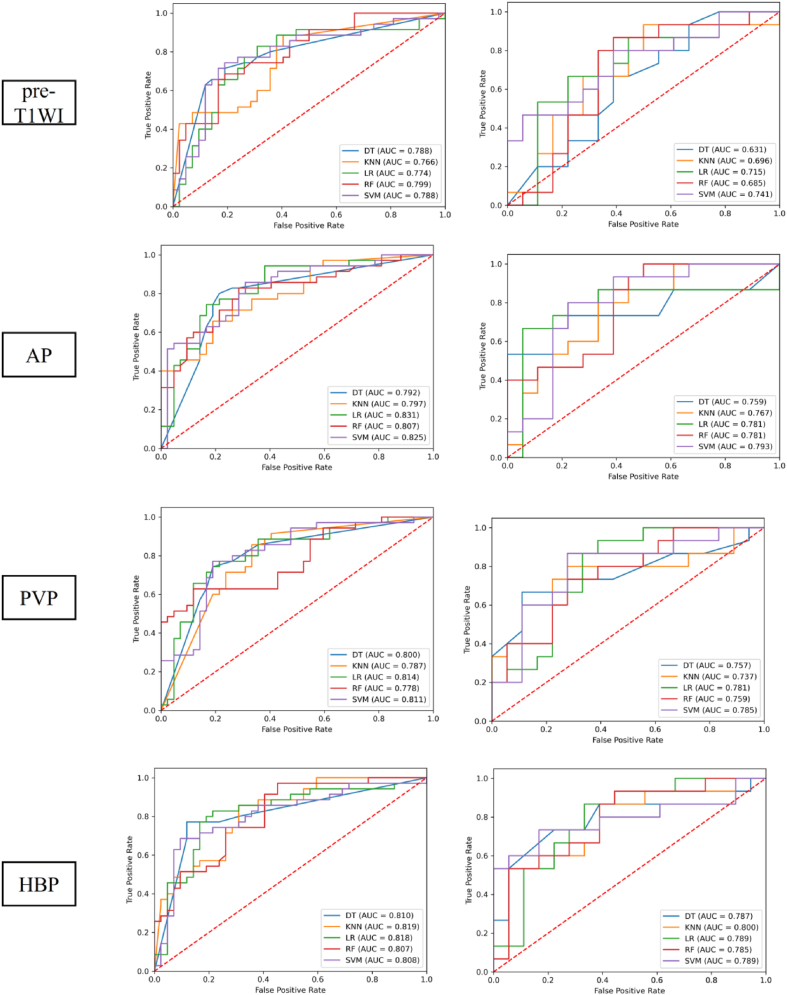
Receiver operating characteristic curves of various models for predicting microvascular invasion status in the training and validation cohorts. HBP DT, decision tree; KNN, K-nearest neighbor; LR, logistic regression; RF, random forest; SVM, support vector machine; pre-T1WI, pre-enhanced T1-weighted imaging; AP, arterial phase; HBP, hepatobiliary phase; PVP, portal venous phase; AUC, area under the curve.

Table 3 and Fig. 4 present the performance of single-phase models using multiple classification algorithms to predict MVI status. In both the training and validation cohorts, the AP and HBP phases exhibited higher classification AUCs than the others. In the training cohort, the RF and LR classifiers outperformed the other classifiers in most phases, while the SVM and LR classifiers outperformed the others in the validation cohort. However, the AUCs did not significantly differ between the five classifiers across each phase.

**Table 3:** Performance of single-phase models in predicting microvascular invasion status.

Phases	Classifier	Training cohort (*n* = 77)						Validation cohort (*n* = 33)					
		AUC	Sen	Spe	Acc	Pre	F1	AUC	Sen	Spe	Acc	Pre	F1
Pre-T1WI	DT	0.788	0.714	0.810	0.766	0.758	0.735	0.631	0.667	0.611	0.636	0.588	0.625
	KNN	0.766	0.886	0.595	0.727	0.646	0.747	0.696	0.933	0.500	0.697	0.609	0.737
	LR	0.774	0.829	0.690	0.753	0.690	0.753	0.715	0.667	0.778	0.727	0.714	0.690
	RF	0.799	0.686	0.810	0.753	0.75	0.716	0.685	0.867	0.611	0.727	0.650	0.743
	SVM	0.788	0.743	0.810	0.779	0.765	0.754	0.741	0.800	0.611	0.697	0.632	0.706
AP	DT	0.792	0.800	0.786	0.792	0.757	0.778	0.759	0.533	1.00	0.788	1.00	0.696
	KNN	0.797	0.657	0.810	0.74	0.742	0.697	0.767	0.800	0.667	0.727	0.667	0.727
	LR	0.831	0.743	0.833	0.792	0.788	0.765	0.781	0.667	0.944	0.818	0.909	0.769
	RF	0.807	0.829	0.714	0.766	0.707	0.763	0.781	1.000	0.500	0.727	0.625	0.769
	SVM	0.825	0.857	0.690	0.766	0.698	0.769	0.793	0.800	0.778	0.788	0.75	0.774
PVP	DT	0.800	0.743	0.810	0.779	0.765	0.754	0.757	0.667	0.889	0.788	0.833	0.741
	KNN	0.787	0.857	0.667	0.753	0.682	0.759	0.737	0.800	0.722	0.758	0.706	0.750
	LR	0.814	0.771	0.786	0.779	0.750	0.761	0.781	0.933	0.611	0.758	0.667	0.778
	RF	0.778	0.629	0.881	0.766	0.815	0.710	0.759	0.733	0.722	0.727	0.688	0.710
	SVM	0.786	0.829	0.690	0.753	0.690	0.753	0.785	0.867	0.722	0.788	0.722	0.788
HBP	DT	0.810	0.771	0.881	0.831	0.844	0.806	0.787	0.733	0.778	0.758	0.733	0.733
	KNN	0.819	0.857	0.690	0.766	0.698	0.769	0.800	0.600	0.944	0.788	0.900	0.720
	LR	0.818	0.829	0.786	0.805	0.763	0.795	0.789	0.867	0.667	0.758	0.684	0.765
	RF	0.807	0.971	0.548	0.740	0.642	0.773	0.785	0.933	0.556	0.727	0.636	0.757
	SVM	0.808	0.686	0.905	0.805	0.857	0.762	0.789	0.733	0.833	0.788	0.786	0.759

DT, decision tree; KNN, K-nearest neighbor; LR, logistic regression; RF, random forest; SVM, support vector machine; pre-T1WI, pre-enhanced T1-weighted imaging; AP, arterial phase; HBP, hepatobiliary phase; PVP, portal venous phase; AUC, area under the curve; Sen, sensitivity; Spe, specificity; Acc, accuracy; Pre, precision; F1, F1 score.

### 3.3 Performance of multi-phase models in predicting MVI status

**Fig. 5:**
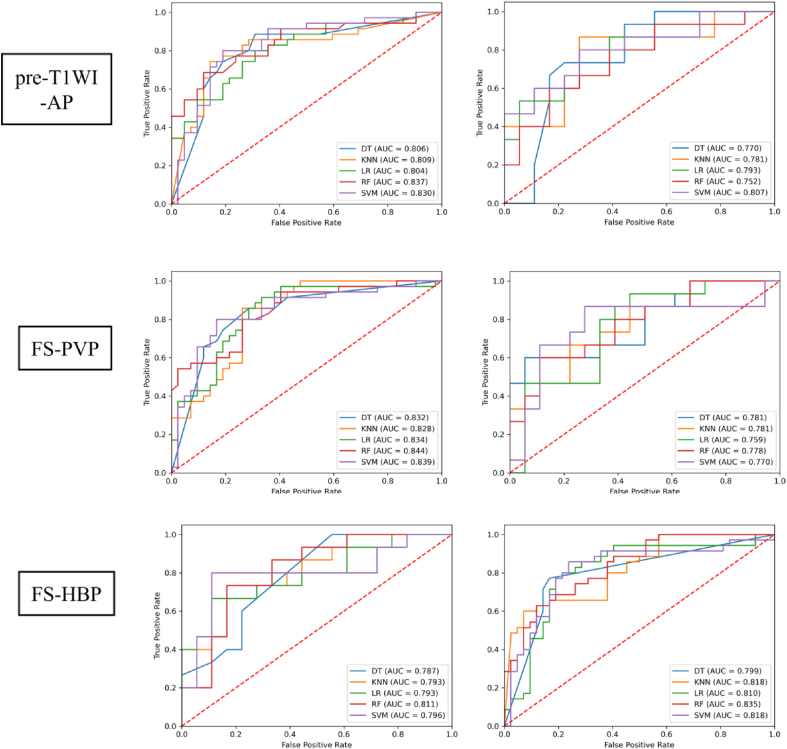
Receiver operating characteristic curves of various models for predicting microvascular invasion status in the training and validation cohorts. pre-T1WI-AP, pre-enhanced T1-weighted imaging + arterial phase; pre-T1WI-PVP, pre-enhanced T1-weighted imaging + portal venous phase; pre-T1WI-HBP, pre-enhanced T1-weighted imaging + hepatobiliary phase; DT, decision tree; KNN, K-nearest neighbor; LR, logistic regression; RF, random forest; SVM, support vector machine; AUC, area under the curve.

**Fig. 6:**
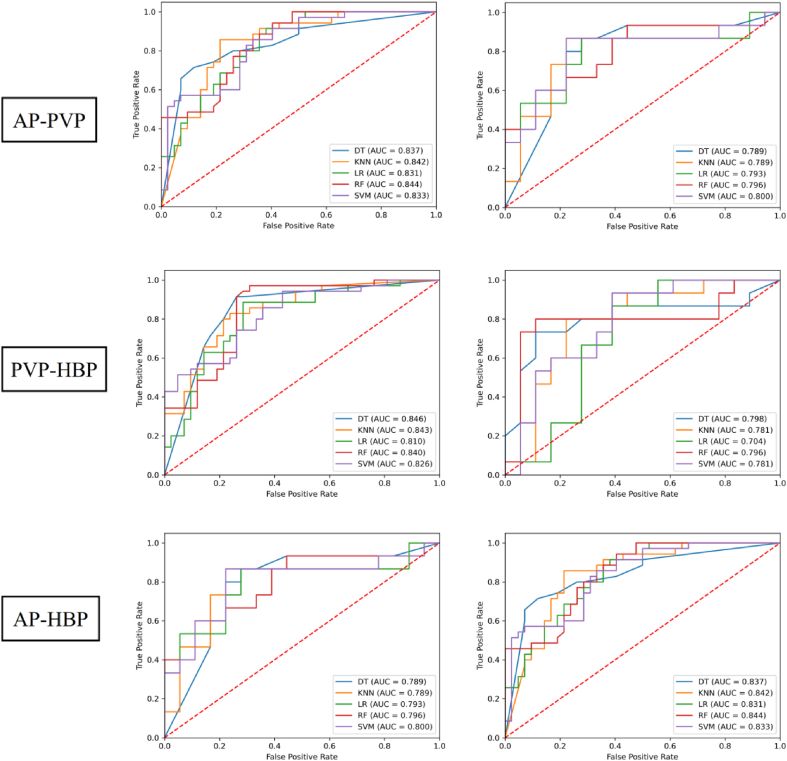
Receiver operating characteristic curves of various models for predicting microvascular invasion status in the training and validation cohorts.HBPHBP AP-PVP, arterial phase + portal venous phase; AP-HBP, arterial phase + hepatobiliary phase; PVP-HBP, portal venous phase + hepatobiliary phase; DT, decision tree; KNN, K-nearest neighbor; LR, logistic regression; RF, random forest; SVM, support vector machine; AUC, area under the curve.

Two-phase models outperformed single-phase models in predicting MVI status (Table 3, Table 4, Table 5 and Fig. 4, Fig. 5, Fig. 6). Notably, the two-phase combination AP-HBP demonstrated the best AUC performance among the other two-phase combinations across all five classifiers in both the training and validation cohorts.

**Table 4:** Performance of two-phase models in predicting microvascular invasion status.

Phases	Classifier	Training cohort (*n* = 77)						Validation cohort (*n* = 33)					
		AUC	Sen	Spe	Acc	Pre	F1	AUC	Sen	Spe	Acc	Pre	F1
Pre-T1WI-AP	DT	0.806	0.886	0.690	0.779	0.705	0.785	0.770	0.733	0.778	0.758	0.733	0.733
	KNN	0.809	0.743	0.857	0.805	0.812	0.776	0.781	0.867	0.722	0.788	0.722	0.788
	LR	0.804	0.800	0.690	0.740	0.683	0.737	0.793	0.867	0.611	0.727	0.650	0.743
	RF	0.837	0.686	0.881	0.792	0.828	0.750	0.752	0.600	0.833	0.727	0.750	0.667
	SVM	0.830	0.800	0.810	0.805	0.778	0.789	0.807	0.800	0.722	0.758	0.706	0.750
FS-PVP	DT	0.832	0.857	0.714	0.779	0.714	0.779	0.781	0.600	0.944	0.788	0.900	0.720
	KNN	0.828	0.857	0.738	0.792	0.732	0.789	0.781	0.933	0.556	0.727	0.636	0.757
	LR	0.834	0.914	0.667	0.779	0.696	0.790	0.759	0.933	0.556	0.727	0.636	0.757
	RF	0.844	0.800	0.738	0.766	0.718	0.757	0.778	0.600	0.889	0.758	0.818	0.692
	SVM	0.839	0.800	0.833	0.818	0.800	0.800	0.770	0.867	0.722	0.788	0.722	0.788
FS-HBP	DT	0.799	0.771	0.833	0.805	0.794	0.783	0.787	1.000	0.444	0.697	0.600	0.750
	KNN	0.818	0.600	0.929	0.779	0.875	0.712	0.793	0.667	0.833	0.758	0.769	0.714
	LR	0.810	0.800	0.786	0.792	0.757	0.778	0.793	0.667	0.889	0.788	0.833	0.741
	RF	0.835	0.629	0.881	0.766	0.815	0.710	0.811	0.733	0.833	0.788	0.786	0.759
	SVM	0.818	0.857	0.762	0.805	0.750	0.800	0.796	0.800	0.889	0.848	0.857	0.828

pre-T1WI-AP, pre-enhanced T1-weighted imaging + arterial phase; pre-T1WI-PVP, pre-enhanced T1-weighted imaging + portal venous phase; pre-T1WI-HBP, pre-enhanced T1-weighted imaging + hepatobiliary phase; DT, decision tree; KNN, K-nearest neighbor; LR, logistic regression; RF, random forest; SVM, support vector machine; AUC, area under the curve; Sen, sensitivity; Spe, specificity; Acc, accuracy; Pre, precision; F1, F1 score.

**Table 5:** Performance of two-phase models in predicting microvascular invasion status.

Phases	Classifier	Training cohort (*n* = 77)						Validation cohort (*n* = 33)					
		AUC	Sen	Spe	Acc	Pre	F1	AUC	Sen	Spe	Acc	Pre	F1
AP-PVP	DT	0.837	0.714	0.881	0.805	0.833	0.769	0.789	0.867	0.722	0.788	0.722	0.788
	KNN	0.842	0.857	0.786	0.818	0.769	0.811	0.789	0.867	0.778	0.818	0.765	0.812
	LR	0.831	0.914	0.619	0.753	0.667	0.771	0.793	0.867	0.722	0.788	0.722	0.788
	RF	0.844	0.943	0.595	0.753	0.660	0.776	0.796	0.933	0.556	0.727	0.636	0.757
	SVM	0.833	0.857	0.667	0.753	0.682	0.759	0.800	0.867	0.778	0.818	0.765	0.812
AP-HBP	DT	0.832	0.800	0.857	0.831	0.824	0.812	0.809	0.667	0.889	0.788	0.833	0.741
	KNN	0.832	0.714	0.857	0.792	0.806	0.758	0.796	1.000	0.611	0.788	0.682	0.811
	LR	0.827	0.886	0.714	0.792	0.721	0.795	0.815	0.867	0.833	0.848	0.812	0.839
	RF	0.821	0.743	0.833	0.792	0.788	0.765	0.793	0.800	0.722	0.758	0.706	0.750
	SVM	0.823	0.800	0.786	0.792	0.757	0.778	0.800	1.000	0.556	0.758	0.652	0.789
PVP-HBP	DT	0.837	0.714	0.881	0.805	0.833	0.769	0.789	0.867	0.722	0.788	0.722	0.788
	KNN	0.842	0.857	0.786	0.818	0.769	0.811	0.789	0.867	0.778	0.818	0.765	0.812
	LR	0.831	0.914	0.619	0.753	0.667	0.771	0.793	0.867	0.722	0.788	0.722	0.788
	RF	0.844	0.943	0.595	0.753	0.660	0.776	0.796	0.933	0.556	0.727	0.636	0.757
	SVM	0.833	0.857	0.667	0.753	0.682	0.759	0.800	0.867	0.778	0.818	0.765	0.812

AP-PVP, arterial phase + portal venous phase; AP-HBP, arterial phase + hepatobiliary phase; PVP-HBP, portal venous phase + hepatobiliary phase; DT, decision tree; KNN, K-nearest neighbor; LR, logistic regression; RF, random forest; SVM, support vector machine; AUC, area under the curve; Sen, sensitivity; Spe, specificity; Acc, accuracy; Pre, precision; F1, F1 score.

**Fig. 7:**
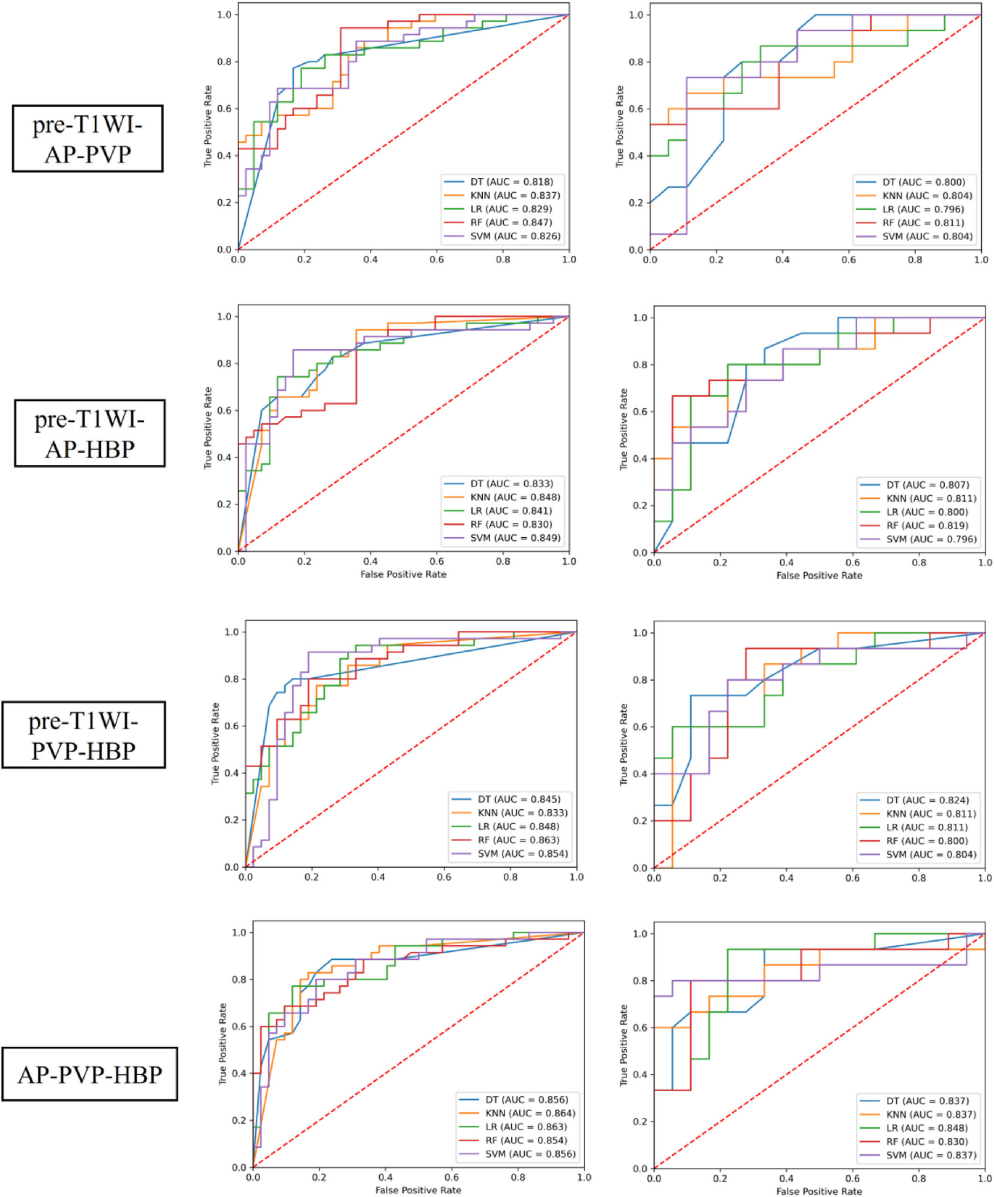
Receiver operating characteristic curves of various models for predicting microvascular invasion status in the training and validation cohorts.HBPHBP AP-PVP-HBP, Pre-enhanced T1-weighted imaging + arterial phase + portal venous phase; preE-T1WI-AP-HBP, pre-enhanced T1-weighted imaging + arterial phase + hepatobiliary phase; pre-T1WI-PVP-HBP, pre-enhanced T1-weighted imaging + portal venous phase + hepatobiliary phase; AP-PVP-HBP, arterial phase + portal venous phase + hepatobiliary phase; DT, decision tree; KNN, K-nearest neighbor; LR, logistic regression; RF, random forest; SVM, support vector machine; AUC, area under the curve.

**Fig. 8:**
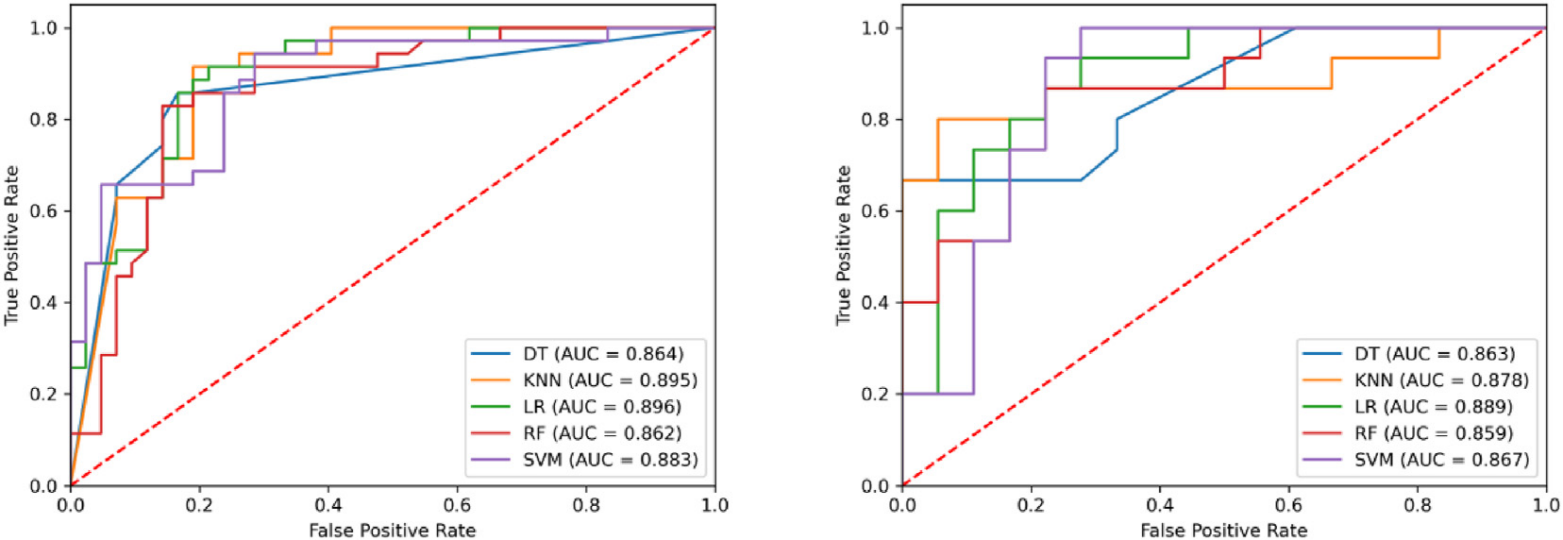
Receiver operating characteristic curves of the pre-T1WI-AP-PVP-HBP model for predicting microvascular invasion status in the training and validation cohortsHBP. pre-T1WI-AP-PVP-HBP, pre-enhanced T1-weighted imaging + arterial phase + portal venous phase + hepatobiliary phase; DT, decision tree; KNN, K-nearest neighbor; LR, logistic regression; RF, random forest; SVM, support vector machine; AUC, area under the curve.

The performance of three-phase models in predicting MVI status was superior to that of the two-phase models (Table 4, Table 5, Table 6 and Fig. 5, Fig. 6, Fig. 7). Among the four three-phase combinations, the AP-PVP-HBP combination achieved the highest AUC in the validation cohort. The four-phase models outperformed three-phase models across all five classifiers in both the training and validation cohorts (Table 6, Table 7 and Fig. 7, Fig. 8). The LR classifier exhibited the best overall performance among the five classifiers, with an AUC of 0.896 in the training cohort and 0.889 in the validation cohort.

**Table 6:** Performance of three-phase models in predicting microvascular invasion status.

Phases	Classifier	Training cohort (*n* = 77)						Validation cohort (*n* = 33)					
		AUC	Sen	Spe	Acc	Pre	F1	AUC	Sen	Spe	Acc	Pre	F1
Pre-T1WI-AP-PVP	DT	0.818	0.771	0.833	0.805	0.794	0.783	0.800	0.800	0.722	0.758	0.706	0.750
	KNN	0.837	0.886	0.619	0.74	0.66	0.756	0.804	0.667	0.889	0.788	0.833	0.741
	LR	0.829	0.771	0.810	0.792	0.771	0.771	0.796	0.867	0.667	0.758	0.684	0.765
	RF	0.847	0.943	0.690	0.805	0.717	0.815	0.811	0.533	1.000	0.788	1.000	0.696
	SVM	0.826	0.686	0.881	0.792	0.828	0.75	0.804	0.733	0.889	0.818	0.846	0.786
Pre-T1WI-AP-HBP	DT	0.833	0.829	0.714	0.766	0.707	0.763	0.807	0.867	0.667	0.758	0.684	0.765
	KNN	0.848	0.943	0.643	0.779	0.688	0.795	0.811	0.800	0.778	0.788	0.750	0.774
	LR	0.841	0.743	0.881	0.818	0.839	0.788	0.800	0.800	0.778	0.788	0.750	0.774
	RF	0.830	0.914	0.619	0.753	0.667	0.771	0.819	0.667	0.944	0.818	0.909	0.769
	SVM	0.849	0.857	0.833	0.844	0.811	0.833	0.796	0.867	0.611	0.727	0.650	0.743
FS-PVP-HBP	DT	0.845	0.800	0.857	0.831	0.824	0.812	0.824	0.733	0.889	0.818	0.846	0.786
	KNN	0.833	0.771	0.786	0.779	0.750	0.761	0.811	0.600	0.944	0.788	0.900	0.720
	LR	0.848	0.943	0.667	0.792	0.702	0.805	0.811	0.600	0.944	0.788	0.900	0.720
	RF	0.863	0.800	0.810	0.805	0.778	0.789	0.800	0.933	0.722	0.818	0.737	0.824
	SVM	0.854	0.914	0.810	0.857	0.800	0.853	0.804	0.800	0.778	0.788	0.750	0.774
AP-PVP-HBP	DT	0.856	0.886	0.762	0.818	0.756	0.816	0.837	0.933	0.667	0.788	0.700	0.800
	KNN	0.864	0.829	0.833	0.831	0.806	0.817	0.837	0.600	1.000	0.818	1.000	0.750
	LR	0.863	0.771	0.881	0.831	0.844	0.806	0.848	0.933	0.778	0.848	0.778	0.848
	RF	0.854	0.686	0.905	0.805	0.857	0.762	0.830	0.800	0.889	0.848	0.857	0.828
	SVM	0.856	0.800	0.810	0.805	0.778	0.789	0.837	0.800	0.944	0.879	0.923	0.857

pre-T1WI-AP-PVP, pre-enhanced T1-weighted imaging + arterial phase + portal venous phase; pre-T1WI-AP-HBP, pre-enhanced T1-weighted imaging + arterial phase + hepatobiliary phase; pre-T1WI-PVP-HBP, pre-enhanced T1-weighted imaging + portal venous phase + hepatobiliary phase; AP-PVP-HBP, arterial phase + portal venous phase + hepatobiliary phase; DT,decision tree; KNN, K-nearest neighbor; LR, logistic regression; RF, random forest; SVM, support vector machine; AUC, area under the curve; Sen, sensitivity; Spe, specificity; Acc, accuracy; Pre, precision; F1, F1 score.

**Table 7:** Performance of the pre-T1WI-AP-PVP-HBP model in predicting microvascular invasion status.

Phases	Classifier	Training cohort (*n* = 77)						Validation cohort (*n* = 33)					
		AUC	Sen	Spe	Acc	Pre	F1	AUC	Sen	Spe	Acc	Pre	F1
Pre-T1WI -AP-PVP-HBP	DT	0.864	0.857	0.833	0.844	0.811	0.833	0.863	0.667	1.000	0.848	1.000	0.800
	KNN	0.895	0.914	0.810	0.857	0.800	0.853	0.878	0.800	0.944	0.879	0.923	0.857
	LR	0.896	0.914	0.786	0.844	0.780	0.842	0.889	0.933	0.722	0.818	0.737	0.824
	RF	0.862	0.829	0.857	0.844	0.829	0.829	0.859	0.867	0.778	0.818	0.765	0.812
	SVM	0.883	0.943	0.714	0.818	0.733	0.825	0.867	1.000	0.722	0.848	0.750	0.857

pre-T1WI-AP-PVP-HBP, pre-enhanced T1-weighted imaging + arterial phase + portal venous phase + hepatobiliary phase; DT, decision tree; KNN, K-nearest neighbor; LR, logistic regression; RF, random forest; SVM, support vector machine; AUC, area under the curve; Sen, sensitivity; Spe, specificity; Acc, accuracy; Pre, precision; F1, F1 score.

Statistical analysis of classifier performance revealed significant advantages of the four-phase model over simpler combinations (Table 7, Table 8). Comparative analysis demonstrated statistically significant superiority of the four-phase model over the two-phase (AP-HBP) and single-phase (HBP) combinations across the SVM, RF, and LR classifiers in MVI prediction. The four-phase model consistently achieved higher 95% confidence intervals (CIs) across all tested classifiers: SVM analysis (0.784–0.959 vs. 0.583–0.827 for AP-HBP and 0.601–0.841 for HBP), RF analysis (0.767–0.950 vs. 0.584–0.828 for HBP), and LR analysis (0.792–0.963 vs. 0.608–0.846 for HBP). Notably, when evaluating the performance across all five classifiers, both the four- and three-phase models exhibited robust predictive capabilities for MVI status, with no statistically significant differences observed between these models across four classifiers in both the training and validation cohorts, suggesting potential redundancy in the additional phase.

**Table 8:** Area under the receiver operating characteristic curve values for the best-performing multi-phase model combinations.

Classifier	pre-T1WI-AP-PVP-HBP		AP-PVP-HBP				AP-HBP				HBP			
Cohorts	TC	VC	TC	P-TC	VC	P-VC	TC	P-TC	VC	P-VC	TC	P-TC	VC	P-VC
DT	0.864	0.863	0.856	0.876	0.837	0.795	0.832	0.091	0.809	0.659	0.810	0.377	0.787	0.535
KNN	0.895	0.878	0.864	0.602	0.837	0.852	0.832	0.292	0.796	0.272	0.819	0.190	0.800	0.456
LR	0.896	0.889	0.863	0.563	0.848	0.788	0.827	0.237	0.815	0.517	0.818	0.236	0.789	**0.031**∗
RF	0.862	0.859	0.854	0.907	0.830	0.936	0.821	0.552	0.793	0.286	0.807	0.421	0.785	**0.045**∗
SVM	0.883	0.867	0.856	0.632	0.837	0.126	0.823	0.284	0.800	**0.028**∗	0.808	0.279	0.789	**0.049**∗

P, statistically significant results from the ROC analysis as compared with pre-T1WI-AP-PVP-HBP; pre-T1WI-AP-PVP-HBP, pre-enhanced T1-weighted imaging + arterial phase + portal venous phase + hepatobiliary phase; AP-PVP-HBP, arterial phase + portal venous phase + hepatobiliary phase; AP-HBP, arterial phase + hepatobiliary phase; HBP, hepatobiliary phase; TC, training cohort; VC, validation cohort; DT, decision tree; KNN, K-nearest neighbor; LR, logistic regression; RF, random forest; SVM, support vector machine; T1WI, Pre-enhanced T1-weighted imaging; AP, arterial phase; HBP, hepatobiliary phase; PVP, portal venous phase; AUC, area under the curve; Sen, sensitivity; Spe, specificity; Acc, accuracy; Pre, precision; F1, F1 score.

Note: Values in bold indicate statistically significant differences (∗: *p* < 0.05).

**Fig. 9:**
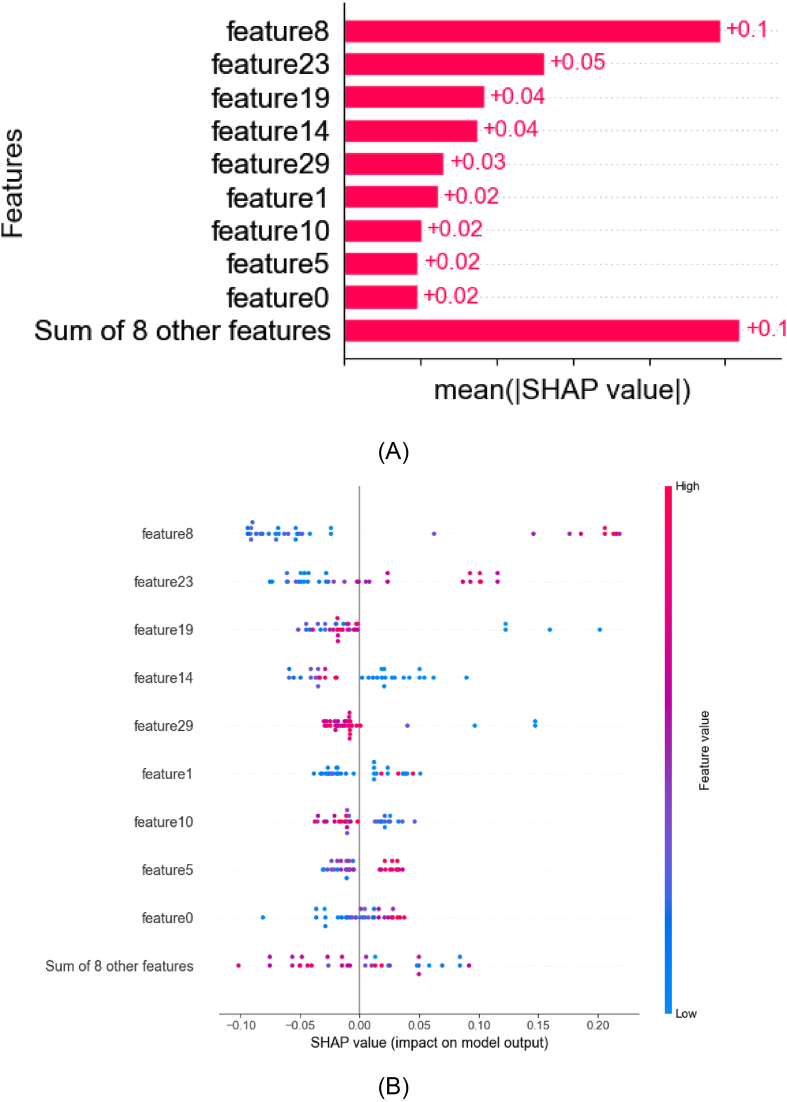
SHAP visualization of global feature importance (A) and SHAP global colony plot by the four-phase model using the RF model (B).

The global feature importance plot (Fig. 9A) highlights that multi-scale radiomic features dominated model predictions. Notably, log-sigma-1-0-mm-3D_glcm_MaximumProbability_AP (feature8), quantifying the concentration of gray-level co-occurrence patterns, exhibited the highest mean SHAP value (+0.1), underscoring its critical role in capturing tumor spatial homogeneity. Morphological indicators such as original_firstorder_Minimum_HBP (feature19) and wavelet-LHH_firstorder_Maximum_HBP (feature23) ranked among the top contributors, with SHAP values of +0.04 and + 0.05, respectively. These features align with biological interpretations: feature19 (lowest intensity in original images) may reflect necrotic regions, while feature23 (maximum intensity in wavelet-transformed images) likely encodes high-frequency structural variations linked to microvascular invasion. Further details about the selected feature are provided in the Supplementary Materials.

The SHAP swarm plot (Fig. 9B) further clarifies directional impacts. High values of feature8 (red clusters at SHAP >0.1) strongly drove positive predictions, suggesting tumors with concentrated texture patterns are associated with higher risk. Conversely, original_glrlm_LongRunLowGrayLevelEmphasis_AP (feature1), despite its lower mean SHAP value (+0.02), showed a bipolar distribution: low feature values (blue) correlated with reduced risk, whereas high values (red) weakly amplified predictions, reflecting its role in encoding heterogeneous run-length distributions. Nonlinear relationships emerged in aggregated features like Sum of 8 other features, where dispersed SHAP values (−0.1 to +0.2) indicated combinatorial effects of texture and intensity metrics.

Importantly, wavelet-derived features demonstrated color-axis coherence: wavelet-LLH_firstorder_RobustMeanAbsoluteDeviation_VP (feature12) and exhibited overlapping SHAP distributions but inverse color gradients, highlighting their complementary roles in quantifying tumor heterogeneity through intensity deviation and low-gray-level dominance. The stability of key features like feature8 and feature23 across cross-validation folds (error bar magnitude <0.01) further validated their robustness, while weaker contributors (e.g., feature5, SHAP +0.02) were retained for marginal predictive synergies. This interpretable framework bridges radiomic signatures to pathophysiological mechanisms, emphasizing the superiority of multi-scale texture analysis over conventional imaging assessments.

**Fig. 10:**
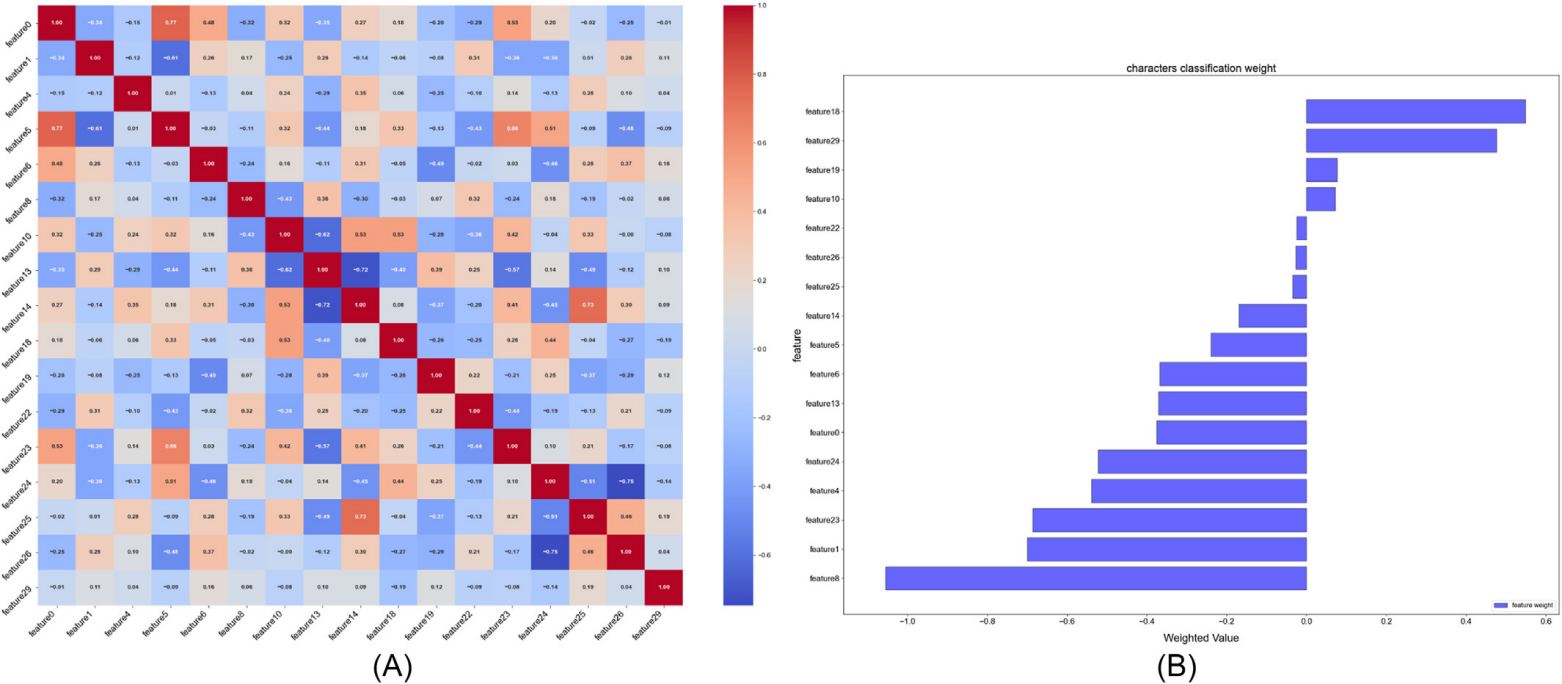

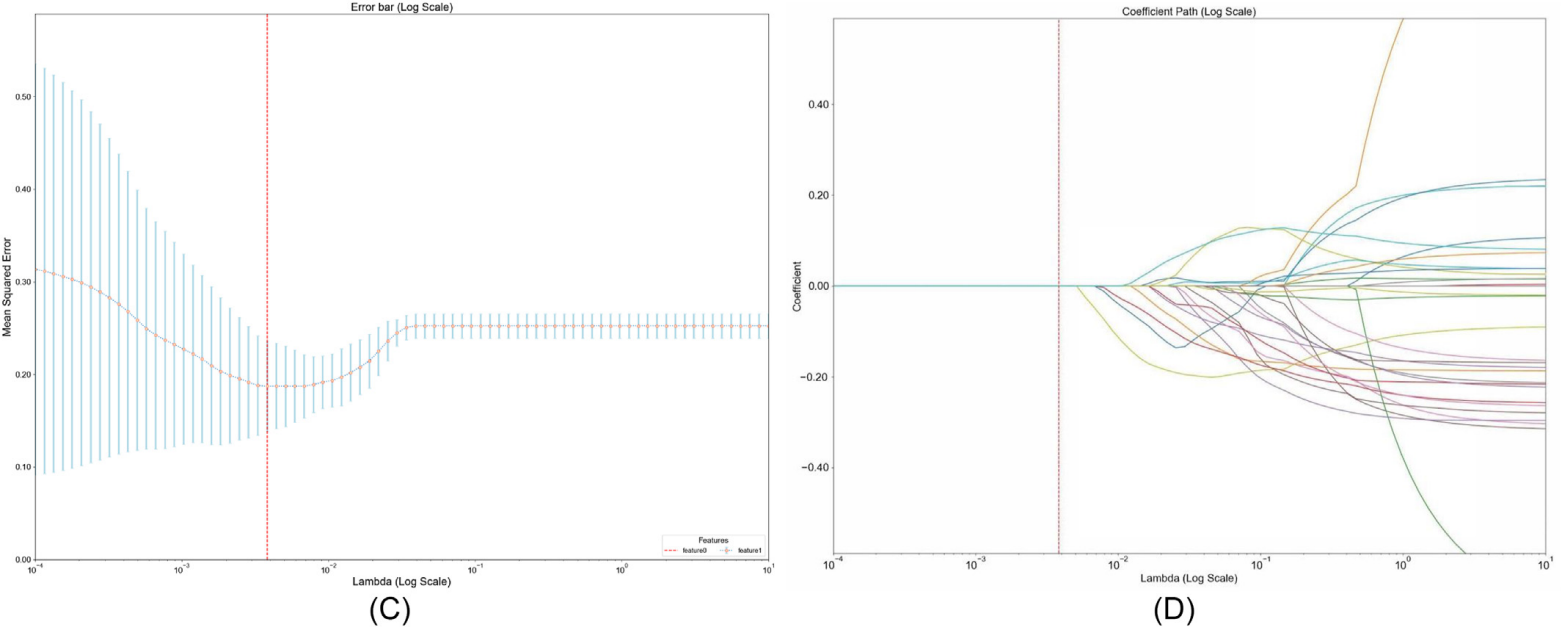
LASSO regression analysis for feature selection. (A) Feature correlation heatmap. (B) LASSO coefficient trajectories across lambda values. (C) Cross-validated mean squared error vs. lambda. (D) LASSO-selected feature coefficients with error bars.

Based on the LASSO regression analysis (Fig. 10D) and cross-validation results (Fig. 10C), the contributions of radiomic features to predictive modeling were systematically evaluated. The classification weight plot (Fig. 10B) revealed distinct feature importance patterns, with log-sigma-5-0-mm-3D_firstorder_90Percentile_VP (feature18, coefficient = 0.549) showing the strongest positive association, while log-sigma-1-0-mm-3D_glcm_MaximumProbability_AP (feature8, coefficient = −1.055) demonstrated significant negative weighting, suggesting its role as a suppressor in the model. The correlation heatmap (Fig. 10A) identified strong multicollinearity among wavelet-derived features, particularly between wavelet-LLH_firstorder_RobustMeanAbsoluteDeviation_VP (feature12) and wavelet-LHH_firstorder_Minimum_VP (feature13) (r = 0.75). LASSO regularization effectively addressed this redundancy by retaining only non-redundant predictors, as evidenced by the coefficient path plot where 13/30 features were shrunk to zero at the optimal lambda (λ = 0.0038). Key texture features survived variable selection, including wavelet-LLL_firstorder_Kurtosis_AP (feature4, −0.539) and original_glrlm_LongRunLowGrayLevelEmphasis_AP (feature1, −0.700), which capture tumor heterogeneity through run-length and wavelet statistics. The error bar plot demonstrated stable model performance (min MSE = 0.187 ± 0.013 at optimal λ), with MSE plateauing when λ > 0.2, indicating reduced overfitting risk. Notably, the selected features encompassed multi-scale characteristics from original (feature1/19/20), wavelet-transformed (feature4-7/12–14/21-27), and log-sigma filtered images (feature8/18/28/29), reflecting complementary biological information. The sparse solution preserved features quantifying tumor texture (glcm/gldm), size heterogeneity (glszm), and intensity distribution (firstorder), aligning with radiomics' emphasis on quantifiable tumor phenotype characteristics. This parsimonious model achieved effective balance between complexity and generalizability through L1 regularization. The LASSO regression results aligned with SHAP interpretability analysis, consistently identifying multi-scale radiomic features as dominant predictors. Key wavelet-based features, such as log-sigma-1-0-mm-3D_glcm_MaximumProbability_AP (feature8), demonstrated robust importance in both frameworks. Despite its negative coefficient in LASSO (−1.055), SHAP analysis revealed that higher values of feature8 (red clusters, SHAP >0.1) strongly drove positive model predictions, suggesting a nuanced role in encoding tumor spatial homogeneity through maximum probability metrics. Similarly, morphological indicators like original_firstorder_Minimum_HBP (feature19) and wavelet-LHH_firstorder_Maximum_HBP (feature23), selected by LASSO (coefficients +0.077 and −0.686, respectively), exhibited significant SHAP contributions (+0.04 and +0.05, respectively), linking low-intensity necrotic regions and wavelet-derived peak intensities to biological risk stratification.

SHAP dependency plots further clarified directional impacts: while feature23 (wavelet-LHH_firstorder_Maximum_HSP) showed a bipolar distribution (high values increasing risk), aggregated features like Sum of 8 other features spanned −0.1 to +0.2 SHAP values, reflecting combinatorial effects of heterogeneous radiomic signatures. The LASSO regularization effectively mitigated multicollinearity observed in wavelet-derived clusters (e.g., between feature18 and feature29), retaining non-redundant predictors such as original_glrlm_LongRunLowGrayLevelEmphasis_AP (feature1), whose SHAP impact (−0.02 to +0.02) aligned with its role in quantifying tumor textural irregularity.

These results validate the clinical utility of integrating radiomics with feature selection methodologies. The stability of feature8 and feature23 across cross-validation folds (error bars <0.01) underscores their reliability in decoding tumor phenotypes, while SHAP's nonlinear insights complement LASSO's linear sparsity. This unified framework highlights the necessity of multi-scale radiomic analysis for preoperative risk modeling, bridging quantitative imaging biomarkers to pathological mechanisms.

## 4 Discussion

In this study, we developed and validated a multi-phase MRI radiomics model for preoperative MVI prediction in HCC. It demonstrated superior performance (AUC = 0.898) compared with the single-phase, two-phase, and three-phase approaches. By analyzing radiomics features extracted from non-contrast, arterial, portal, and hepatobiliary phases, the integrated model effectively captured complementary imaging characteristics unique to each phase. This highlights the importance of leveraging multi-phase data to encapsulate the complex interplay between tumor morphology, vascularity, and perfusion dynamics that underpin MVI.

Among the five machine learning classifiers evaluated, the logistic regression classifier consistently delivered optimal performance, achieving robust results in both the training (AUC = 0.898; 95% CI, 0.792–0.963) and validation cohorts (AUC = 0.889; 95% CI, 0.781–0.982). The progressive improvement in predictive accuracy from single-phase (AUC = 0.789) in the validation cohort by the LR model to multi-phase models underscores the critical value of integrating complementary information across contrast-enhanced phases. These results align with the pathophysiological nature of MVI, which reflects heterogeneous tumor vascularity and perfusion patterns manifesting at distinct temporal stages during contrast enhancement. By capturing these subtle variations, the multi-phase approach offers a more comprehensive and nuanced assessment of MVI risk.

Our findings provide promising insights into improving the preoperative evaluation of HCC patients, with potential implications for refining treatment strategies. For instance, more accurate MVI prediction could aid in tailoring surgical margins, guiding the decision between liver transplantation and resection, and identifying candidates for adjuvant therapy. Furthermore, the superior performance of the logistic regression classifier suggests that simpler, interpretable models may be sufficient for integrating radiomics features in clinical workflows, thus enhancing the practical applicability of our approach.

Despite these strengths, several study limitations warrant consideration. First, the single-center retrospective design and relatively small sample size may constrain the generalizability of our findings. Future multi-center studies with larger, more diverse cohorts are essential to validate the robustness and reproducibility of the model across different populations and imaging protocols. Second, potential selection bias in patient recruitment may have influenced the observed outcomes, highlighting the need for more representative sampling strategies in future research. Third, the lack of standardization in image acquisition and processing across institutions presents a technical barrier to broader implementation. Addressing these challenges will require collaborative efforts to establish uniform imaging protocols and quality control measures.

Additionally, our study primarily focused on the radiomics features extracted from static imaging data. Future research could explore the integration of dynamic imaging biomarkers, such as temporal changes in contrast enhancement, with radiomics features to further enhance MVI prediction. The inclusion of advanced deep learning models could also offer opportunities to automatically extract hierarchical features, potentially improving predictive performance without manual feature selection. Lastly, prospective studies are needed to assess the clinical utility of the proposed model in real-world settings, particularly its impact on decision-making and patient outcomes.

## 5 Conclusion

Our study demonstrates that integrating multi-phase MRI radiomics features significantly enhances the prediction of MVI in HCC patients. The proposed model, with its superior performance, holds promise as a non-invasive tool for preoperative risk stratification, providing valuable insights to clinicians for optimizing treatment planning and decision-making. By leveraging complementary information from non-contrast, arterial, portal, and hepatobiliary phases, this multi-phase integration strategy effectively captures the intricate vascular and perfusion characteristics of HCC, offering a more nuanced and comprehensive assessment of tumor biological behavior.

The potential clinical implications of this approach are substantial. Accurate preoperative prediction of MVI can guide tailored surgical strategies, inform the selection between liver transplantation and resection, and identify candidates for adjuvant therapies, ultimately improving patient outcomes. Furthermore, the model's reliance on interpretable radiomics features and logistic regression makes it both practical and accessible for integration into clinical workflows.

However, translating this model into routine practice will require further validation. Prospective, multi-center studies with larger, more diverse patient cohorts are essential to confirm its robustness and generalizability. Addressing technical challenges, such as standardizing imaging protocols and radiomics feature extraction across institutions, will also be critical for facilitating widespread adoption. Exploring the integration of dynamic imaging biomarkers and advanced machine learning techniques could further enhance the model's predictive capabilities.

## CRediT authorship contribution statement

**Yue Peng:** Writing – review & editing, Writing – original draft, Visualization, Methodology, Formal analysis, Conceptualization. **Songxiong Wu:** Validation, Software, Methodology, Data curation, Conceptualization. **Bing Xiong:** Formal analysis. **Fuqiang Chen:** Visualization, Investigation. **Nazar Zaki:** Writing – review & editing. **Ruodai Wu:** Writing – original draft, Funding acquisition. **Wenjian Qin:** Writing – review & editing, Writing – original draft, Resources, Funding acquisition, Conceptualization.

## Informed consent

It was waived by the Medical Ethics Committee of Shenzhen University General Hospital (approval no. KYLLMS-2021-13).

## Organ donation

Not applicable.

## Ethical approval

This study was granted an exemption from ethical review by the Medical Ethics Committee of Shenzhen University General Hospital (approval no. KYLLMS-2021-13). The research did not involve human participants, animal experiments, or sensitive personal data requiring direct ethical oversight. All analyzed data were anonymized and complied with institutional and national guidelines for research integrity.

## Data availability statement

The data that support the conclusions of this study can be made available by the corresponding author upon reasonable request.

## Animal treatment

Not applicable.

## Declaration of Generative AI and AI-assisted technologies in the writing process

Not applicable.

## Funding

The study was funded by Shenzhen Municipal Scheme for Basic Research (No. JCYJ20210324100208022).

## Declaration of competing interest

The authors and funders declare no conflict of interest.
