# Supplementary material for: Multiphase MRI radiomics model for predicting microvascular invasion in HCC: Development and clinical validation
Source: ILIVER. 2025 Apr 26;4(2):100165. doi: 10.1016/j.iliver.2025.100165 (PMC12209474; doi:10.1016/j.iliver.2025.100165)
Supplement: Multimedia component 1 [file mmc1.docx]

**Supplementary S1.**

**Part 1. Radiomic Feature Extraction**

We analyzed the radiomic features by adopting the V3.0.1 version of Pyradiomics. The images were normalized with a normalization ratio of 50, and the BSpline interpolator method was used to resample all images to the average spacing of training set. Radiomic features we extracted included first order statistics, shape-based (2D and 3D), Gray Level cooccurence matrix (GLCM), Gray Level Size Zone Matrix (GLSZM), Gray Level Run Length Matrix (GLRLM), Neighbouring Gray Tone Difference Matrix (NGTDM), Gray Level Dependence Matrix (GLDM). All the above features were extracted using the default settings. In addition, there are Laplacian of Gaussianfiltered (with Sigma values 1.0, 3.0, 5.0), wavelet-decomposition-based (using the coiflet 1 function). A specific description of the feature can be found in the Pyradiomics document. For each patient, we extracted 1130 features each from each imaging phase (pre-T1WI, AP, PVP, and HBP).

This is the PyRadiomics configuration for the radiomics experiment setup in this study:

# Settings to use, possible settings are listed in the documentation (section "Customizing the extraction").

setting:

normalize: True

normalizeScale: 50

binWidth: 25

label: 1

interpolator: 'sitkBSpline'

resampledPixelSpacing: [x_average spacing, y_average spacing, z_average spacing]

imageType:

Original: {}

Wavelet: {}

LoG:

sigma: [1., 3., 5.]

featureClass:

shape: # all shape

firstorder: # specifying an empty list has the same effect as specifying nothing.

glcm: # Disable SumAverage by specifying all other GLCM features available

glrlm: # for lists none values are allowed, in this case, all features are enabled

glszm:

gldm: # contains deprecated features, but as no individual features are specified, the deprecated features are not enabled

ngtdm:

voxelSetting:

kernelRadius: 2

maskedKernel: true

initValue: 0

voxelBatch: 10000

**Part 2. Feature Selection**

We initiated the process by calculating the F-values between the labels and features using the f_classif function from the feature selection library. Subsequently, we employed the SelectKBest function from the same library with k set to 100 to select the top 100 features. Following this, we calculated the chi-squared values for the features using the chi2 function from the feature selection library and applied SelectKBest again, this time with k=30, to further narrow down the features to the top 30. Finally, we refined these 30 features using the Least Absolute Shrinkage and Selection Operator (LASSO) with cross-validation. LASSO, implemented via LassoCV from scikit-learn, integrates L1 regularization into its loss function to manage model complexity and automatically selects features by shrinking the coefficients of irrelevant features to zero. The optimal regularization parameter (alpha) was determined through 10-fold cross-validation and only features with non-zero coefficients were retained in the final feature set. The regularization path was systematically constructed using np.logspace(-4, 4, 300) to generate 300 α values spanning four orders of magnitude, ensuring comprehensive coverage of potential regularization intensities from minimal shrinkage to complete feature elimination. This configuration adheres to the CRISP-ML standard for hyperparameter optimization in high-dimensional radiomic studies.

**Part 3. Model Construction**

The study group was randomly divided into training and validation cohorts at a ratio of 7:3. The training cohort was standardized using the preprocessing.StandardScaler() function, and the validation cohort was standardized using the parameters derived from the training cohort. The hyperparameters for each classifier used in the model training were set as follows:

def class_para(self):

# SVM

self.svm_kernel = ['rbf', 'sigmoid']

# self.svm_kernel = ['linear', 'poly', 'rbf', 'sigmoid']

self.svm_C = [1e-8, 1e-6, 1e-5, 1e-3, 1e-2, 1e-1, 1, 32, 100, 200, 300, 400, 1000]

self.svm_Gamma = [1e-8, 1e-6, 1e-5, 1e-3, 1e-2, 1e-1]

self.svm_classwight = [{0: 0.55, 1: 1}, {0: 0.6, 1: 1}, {0: 0.65, 1: 1}, {0: 0.7, 1: 1}, {0: 0.8, 1: 1},{0: 1, 1: 1}]

self.svm_max_iter = [2, 4, 10, 20, 25, 30, 40, 60, 140, 200, 300, -1]

self.svm_grid_search_param = [{"kernel": self.svm_kernel, "C": self.svm_C, "gamma": self.svm_Gamma, "class_weight": self.svm_classwight, "max_iter": self.svm_max_iter}]

# Logistic

self.lr_penalty = ['l2']

self.lr_C = [0.01, 0.05, 0.1, 0.3, 0.4, 0.5, 0.6, 0.8, 1.0, 1.2]

self.lr_max_iter = [10, 20, 40, 60, 80, 100, 150, 200, 400, 500]

self.lr_tol = [1e-5, 1e-4, 1e-3, 1e-2]

self.lr_grid_search_param = [{"penalty": self.lr_penalty, "C": self.lr_C, "max_iter": self.lr_max_iter, "tol": self.lr_tol}]

#RF

self.rf_n_estimators = [10, 20, 30 , 50 ,60 ,80 , 101, 151, 201, 251, 301, 351, 401, 451, 501, 551, 800]

self.rf_max_depth=[ None ,1, 3, 4, 5, 6, 8, 10, 12, 15, 20]

self.rf_min_samples_split=[1, 3, 5, 7, 9 , 10, 12, 15]

self.rf_min_samples_leaf: [1, 2, 3, 4, 5, 7, 9, 10],

self.rf_grid_search_param = [{"n_estimators": self.rf_n_estimators, " max_depth ": self.rf_max_depth , " min_samples_split ": self.rf_ min_samples_split , " min_samples_leaf ": self.rf_min_samples_leaf }]

# DT

self.dt_criterion = ['gini', 'entropy']

self.dt_max_depth = [None, 5, 8, 10, 12, 15, 17, 20, 25, 30]

self.dt_min_samples_split = [2, 3, 5, 7, 10]

self.dt_min_samples_leaf = [1, 2, 3, 4, 5, 7, 9, 10]

self.dt_grid_search_param = [{"criterion": self.dt_criterion, "max_depth": self.dt_max_depth, "min_samples_split": self.dt_min_samples_split, "min_samples_leaf": self.dt_min_samples_leaf}]

# KNN

self.knn_n_neighbors = [3, 5, 7, 10, 15, 20]

self.knn_weights = ['uniform', 'distance']

self.knn_metric = ['euclidean', 'manhattan']

self.knn_grid_search_param = [{"n_neighbors": self.knn_n_neighbors, "weights": self.knn_weights, "metric": self.knn_metric}]

**Part 4. Final Feature Selection**

The following 17 features were selected through a four-phase LASSO-CV feature selection process.

[fearure0：'original_glcm_ClusterTendency_AP',

fearure1：'original_glrlm_LongRunLowGrayLevelEmphasis_AP',

fearure2：'original_glszm_SmallAreaEmphasis_AP',

fearure3：'wavelet-HLH_glcm_ClusterShade_AP',

fearure4：'wavelet-LLL_firstorder_Kurtosis_AP',

fearure5：'wavelet-LLL_glcm_DifferenceEntropy_AP',

fearure6：'wavelet-LLL_glrlm_RunEntropy_AP',

fearure7：'wavelet-LLL_glszm_LowGrayLevelZoneEmphasis_AP',

fearure8：'log-sigma-1-0-mm-3D_glcm_MaximumProbability_AP',

fearure9：'original_glrlm_RunLengthNonUniformity_VP',

fearure10：'original_glszm_SmallAreaHighGrayLevelEmphasis_VP',

fearure11：'wavelet-LLH_firstorder_MeanAbsoluteDeviation_VP',

fearure12：'wavelet-LLH_firstorder_RobustMeanAbsoluteDeviation_VP',

fearure13：'wavelet-LHH_firstorder_Minimum_VP',

fearure14：'wavelet-HHH_firstorder_Energy_VP',

fearure15：'wavelet-HHH_firstorder_InterquartileRange_VP',

fearure16：'wavelet-LLL_glcm_JointAverage_VP',

fearure17：'wavelet-LLL_glcm_SumAverage_VP',

fearure18：'log-sigma-5-0-mm-3D_firstorder_90Percentile_VP',

fearure19：'original_firstorder_Minimum_HBP',

fearure20：'original_glszm_LargeAreaHighGrayLevelEmphasis_HBP',

fearure21：'wavelet-LLH_glcm_Idn_HBP',

fearure22：'wavelet-LLH_glszm_SmallAreaLowGrayLevelEmphasis_HBP',

fearure23：'wavelet-LHH_firstorder_Maximum_HBP',

fearure24：'wavelet-LHH_gldm_SmallDependenceLowGrayLevelEmphasis_HBP',

fearure25：'wavelet-LHL_glszm_LargeAreaHighGrayLevelEmphasis_FS',

fearure26：'wavelet-HLL_gldm_LargeDependenceEmphasis_FS',

fearure27：'wavelet-HHL_firstorder_Maximum_FS',

fearure28：'log-sigma-1-0-mm-3D_ngtdm_Complexity_FS',

fearure29：'log-sigma-5-0-mm-3D_glcm_Imc2_FS']
